# COVID-19 and a Tale of Three Drugs: To Repurpose, or Not to Repurpose–That Was the Question

**DOI:** 10.3390/v17070881

**Published:** 2025-06-23

**Authors:** Chris R. Triggle, Ross MacDonald

**Affiliations:** Weill Cornell Medicine-Qatar, Doha P.O. Box 24144, Qatar

**Keywords:** COVID-19, repurposing, hydroxychloroquine, ivermectin, remdesivir

## Abstract

On 11 March 2020, the World Health Organisation (WHO) declared a global pandemic caused by the SARS-CoV-2 coronavirus that earlier in February 2020 the WHO had named COVID-19 (coronavirus disease 2019). There were neither drugs nor vaccines that were known to be effective against the virus, stimulating an urgent worldwide search for treatments. An evaluation of existing drugs by ‘repurposing’ was initiated followed by a transition to de novo drug discovery. Repurposing of an already approved drug may accelerate the introduction of that drug into clinical use by circumventing early, including preclinical studies otherwise essential for a de novo drug and reducing development costs. Early in the pandemic three drugs were identified as repurposing candidates for the treatment of COVID-19: (i) hydroxychloroquine, an anti-malarial also used to treat rheumatoid arthritis and lupus; (ii) ivermectin, an antiparasitic approved for both human and veterinary use; (iii) remdesivir, an anti-viral originally developed to treat hepatitis C. The scientific evidence, both for and against the efficacy of these three drugs as treatments for COVID-19, vied with public demand and politicization as unqualified opinions clashed with evidence-based medicine. To quote Hippocrates, “*There are in fact two things, science and opinion; the former begets knowledge, the latter ignorance*”.

## 1. Introduction

So begins the 1859 Charles Dickens classic *A Tale of Two Cities*, “It was the best of times, it was the worst of times, it was the age of wisdom, it was the age of foolishness, it was the epoch of belief, it was the epoch of incredulity, it was the season of Light, it was the season of Darkness…” [1], and so began 2020 with the world suddenly engulfed in a global pandemic with no known treatments available. The world entered a period of near panic where scientific evidence was challenged by misinformation, public pressure and politicization. On 1 January 2020, the Huanan Seafood Market in Wuhan, China, was closed over fears of a virus outbreak, which at the time seemed similar to the 2002 SARS outbreak. On 13 January 2020, Thailand confirmed the first case of COVID-19 outside of China, and the first case in the USA was confirmed by the Centers for Disease Control and Prevention (CDC) on 20 January 2020 in Washington State from a sample that was taken on 18 January 2020 [2]. The first cases in Europe were reported shortly after on 24 January in Bordeaux, France. On 21 January 2020, the Chinese Government confirmed that human-to-human transmission was spreading the virus. The International Committee on Taxonomy of viruses named the virus “severe acute respiratory syndrome coronavirus 2” (SARS-CoV-2) [3]. The WHO declared a pandemic on 11 March 2020 [4]. COVID-19 truly became global in December 2020 when 60 cases were identified in several Chilean stations in Antarctica [5].

The serious issues of how to deal with a global pandemic that would eventually reach even the most remote parts of the planet and could potentially infect the entire human population were obvious and urgent steps were needed. Fortuitously, Dr. Zhang Yong-Zhen, a scientist at Fudan University in Shanghai, sequenced the genome of the offending virus, a coronavirus, which the greater majority of scientific evidence indicates was of zoonotic origins and a strain of the species *Betacoronavirus pandemicum*, and made it available to the entire world via the Chinese Centre for Disease Control and Prevention. This was then released on GISAID (Global Initiative on Sharing All Influenza Data) shortly after midnight GMT on 10 January 2020 [6]. The timely availability of the genome sequence for SARS-CoV-2 enabled the fast-tracking of a number of vaccines, including the novel mRNA vaccines developed by Pfizer-BioNTech and Moderna [7,8]. However, the first vaccines, Sinopharm and CoronaVac, from China, and based on inactivated virus, did not become available for use until July and August 2020, respectively. The Russian vaccine, Sputnik, was released in August 2020 and was based on the use of an adenovirus vector. In December 2020, the Oxford-AstraZeneca vaccine, also using an adenovirus vector, and the two mRNA vaccines from Pfizer-BioNTech and Moderna became available. Drs. Katalin Karikó and Drew Weissman were jointly awarded the 2023 Nobel Prize in Physiology or Medicine for their pioneering research that ultimately led the development of the mRNA vaccines for use against COVID-19. However, what was urgently missing were effective antiviral drugs to treat those already infected with the virus, and antiviral drugs that could be distributed for prophylactic use. Again, a worldwide effort was initiated with one focus being on re-purposing drugs already approved for other diseases.

## 2. The Benefits of Repurposing Drugs

The repurposing (or repositioning) of a drug already approved for another disease has proved beneficial in accelerating and even by-passing several of the pre-clinical, discovery and Phase 1 pharmacokinetic studies, as well as reducing Phase 2 studies that would otherwise be essential for a new drug prior to initiating Phase 3 clinical trials [9]. In addition, the costs of bringing a repurposed drug to market have been estimated as less than one sixth the cost of that of a de novo new chemical entity [9]. Although there are potential disadvantages in repurposing drugs, there are examples of drugs that have been successfully repurposed, including aspirin as an anti-platelet drug, thalidomide for cancer, and sildenafil first for erectile dysfunction and later for pulmonary hypertension [10]. There are different approaches that have been used to identify which drugs may be appropriate for repurposing: one used for identifying antiviral agents for SARS-CoV-2 was based on using the virus’s proteins and their interaction with human proteins as the druggable targets, as based on viral assays. In one study, 29 drugs already approved by the U.S. Food & Drug Administration (FDA) were identified as potentially appropriate for further investigation, which of particular interest to our discussions included hydroxychloroquine [11]. From the perspective of the pharmaceutical industry, a disadvantage of repurposing is the issue of patentability. Another potential disadvantage relates to the dosages required for the new indication that may be higher than originally tested in the Phase 1 trials. Related to dosages is the question of the validity of repurposing a drug based on in vitro data versus the pharmacokinetic properties of the drug when used in humans, and this is of particular relevance when we discuss the use of ivermectin for COVID-19.

In this article we will focus on three drugs that were, in the early stages of the COVID-19 pandemic, the subject of not only intensive investigation for repurposing as antiviral drugs for both prophylaxis and treatment of infection with SARS-CoV-2, but also the subject of considerable controversy. Unfortunately, the controversy over the effectiveness of treatments was heightened by intense media attention, popularization, and politicization, as well as controversial and, arguably, fraudulent scientific data, with evidence of messianism in the promotion of at least one of the treatments as a miracle drug [12,13]. The drugs to be discussed are the anti-malarial and immunosuppressive drug, hydroxychloroquine; the antiparasitic drug, ivermectin; and the antiviral drug, remdesivir. The latter was originally evaluated as an antiviral for hepatitis C and Ebola but not pursued for either disease due to clinically superior alternatives.

## 3. Hydroxychloroquine

There is a long history of the use of the 4-aminoquinoline drugs hydroxychloroquine and chloroquine for the treatment of malaria. Hydroxychloroquine is also used to treat rheumatoid arthritis and lupus. The FDA first approved hydroxychloroquine to treat malaria in 1955, and its extensive use and the depth of knowledge of potential side effects justified the evaluation of the drug for COVID-19 [14]. Furthermore, a 2005 in vitro cell culture study had reported that the chemically related drug, chloroquine possessed antiviral activity against the SARS-CoV virus with an IC_50_ of 1 μM [15], thus suggesting a potential benefit for use in patients with COVID-19. However, as has been stressed for other drugs such as metformin [16], effectiveness based on in vitro studies in non-human cells or in vivo studies in animal models do not always equate to clinical efficacy. A variety of pharmacokinetic issues including bioavailability, tissue distribution, and metabolism can greatly reduce therapeutic efficacy and may enhance toxicity. Arguably, because hydroxychloroquine is a basic drug with a pKa of 9.5 it will be taken up and accumulated into lysosomes where it will raise the pH, thereby preventing the acidification process that is essential for the cellular entry of the coronavirus and was originally proposed to explain the in vitro effectiveness of the 4-aminoquinolines against SARS [15,17,18,19,20]. Indeed, the accumulation of hydroxychloroquine in the acidic environment of the food vacuoles of the malaria parasite is considered to be an important mechanism for the drug’s efficacy as an anti-malarial drug [21]. Furthermore, since hydroxychloroquine has an extremely long elimination half-life of over 30 days this prolonged maintenance of tissue levels of the drug might further enhance its antiviral actions [22].

**Figure 1:**
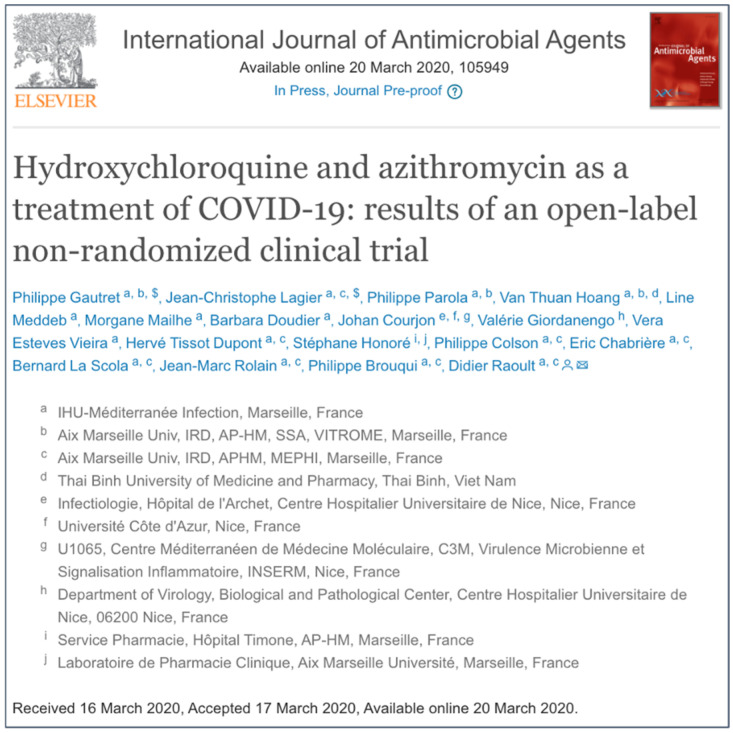
Screenshot showing the peer review and publication dates for Gautret et al. [25] suggesting a rush to publish by both authors and the journal. This paper was retracted on 17 December 2024.

Interestingly, hydroxychloroquine was identified as possessing antiviral activity against SARS-CoV-2, but concern was expressed over the already known off-target effects, including the cardiac hERG potassium channel [11]. These concerns were possibly overlooked or ignored when it was announced by a research group at Aix Marseille University that hydroxychloroquine, and the related anti-malarial drug chloroquine, were effective in humans against COVID-19 [23,24]. On 17 March 2020 after only one day under review (Figure 1), the results from an open-label non-randomized control study from the same group in Marseille were published, reporting that combining hydroxychloroquine with the macrolide antibiotic azithromycin, resulted in a significant reduction in the viral load in 20 patients with symptomatic COVID-19 [25]. This particular drug combination should have raised concerns as azithromycin is a cytochrome P450 (CYP450) inhibitor and hydroxychloroquine, which, as already noted, has a particularly long elimination half-life of over 30 days, is metabolized by CYP450. In consequence, combining hydroxychloroquine with azithromycin raises the risk for precipitating cardiac arrhythmias (as has been reported when the drugs are used individually as monotherapy), and is associated with prolongation of the QT interval, triggering *torsades de pointes* [26,27]. Despite these concerns, the publicity that resulted from this study, including endorsement by President Trump in the USA, resulted in a worldwide surge in the use of hydroxychloroquine to treat COVID-19 [28]. On 26 March 2020 the French Minister of Health quickly approved the use of hydroxychloroquine to treat COVID-19. The FDA in the USA followed and on 28 March 2020 gave emergency authorization for both hydroxychloroquine and chloroquine. A plethora of clinical studies followed but it rapidly became clear that hydroxychloroquine in the clinical setting was ineffective against COVID-19 [29]. Approval was short-lived in both countries: France rescinded approval on 26 May 2020, and the USA followed on 15 June. Other countries also withdrew approval for hydroxychloroquine, although significant interest in the use of the drug against COVID-19 persisted despite scientific concerns over the validity of data and the effectiveness of hydroxychloroquine against COVID-19 [29]. An analysis of the data regarding hydroxychloroquine usage in RCTs for COVID-19, primarily from the RECOVERY and the WHO SOLIDARITY trials, concluded that use increased mortality [30]. Furthermore, the use of hydroxychloroquine in patients with COVID-19 and no cardiovascular comorbidity was associated with prolonged QTc and cardiac arrhythmias, including *torsades de pointes*, whereas in patients treated with hydroxychloroquine for systemic lupus erythematosus and rheumatoid arthritis the use of hydroxychloroquine a reduction in cardiovascular disease is seen [31].

As a result of multiple investigations, in May 2022 the French National Agency for the Safety of Medicines and Health Products announced it would bring charges against the Institut Hospitalo-Universitaire (ICH) in Marseille. Although data from additional studies were published, concerns over study design and methodological issues, combined with ethical questions regarding unrelated prior studies also headed by Dr. Raoult (including to date 10 retractions and more than 100 “expressions of concern”) ultimately resulted in his resignation from his position with the ICH at the AIX-Marseille University [28]. The Gautret et al. study [25] was eventually retracted on 17 December 2024 nearly four and a half years after its original publication [32].

In the USA an 80-fold increase in the prescribing of hydroxychloroquine was reported in March 2020 versus March 2019 [33]. In addition to the potential for patient harm from inappropriate use, the widespread uptake of hydroxychloroquine to treat COVID-19 also resulted in a shortage of the drug to treat medical conditions such as lupus, where it has clear benefits [34,35,36]. Similarly, concerns and warnings were issued that the widespread off-label use of hydroxychloroquine for COVID-19 outside of appropriately designed, randomized controlled clinical trials would contribute to a lack of evidence-based data as to the effectiveness of the drug [37,38]. Global data for how many individuals suffered serious side-effects or fatalities due to hydroxychloroquine use for COVID-19 were not found but several reports provide insight. For instance, a study from Italy reported 306 cases and 9 fatalities due to off-label hydroxychloroquine use over the period 1 March 2020 to 31 May 2020, albeit with some concerns over the accuracy of the data in terms of both under-estimating and over-estimating the number of cases [39].

**Figure 2:**
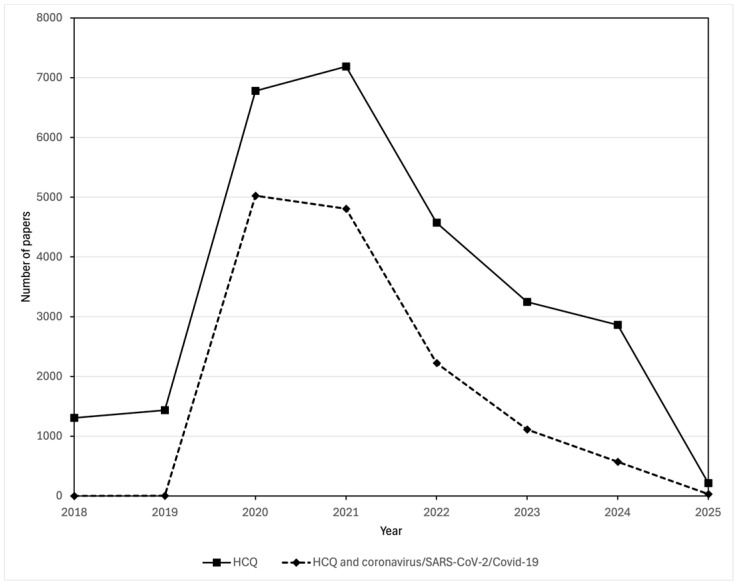
Comparison of the number of publications in the Scopus database about hydroxychloroquine and those about both hydroxychloroquine and coronavirus/SARS-CoV-2/COVID-19. Publications mentioning hydroxychloroquine were found by searching for *(TITLE-ABS-KEY (hydroxychloroquine)) AND (PUBYEAR > 2017)*. Publications mentioning both hydroxychloroquine and coronavirus/SARS-CoV-2/COVID-19 were found by searching for *(TITLE-ABS-KEY (hydroxychloroquine)) AND (TITLE-ABS-KEY (coronavirus OR COVID-19 OR SARS-CoV-1 OR SARS-CoV-2)) AND (PUBYEAR > 2017))*.

Not surprisingly, research interest in the use of hydroxychloroquine against COVID-19 or coronaviruses in general spiked early in the pandemic, as is reflected in the SCOPUS data for the number of papers published (Figure 2). However, the interest persisted and was enhanced by inappropriate media attention, politization, and, surprisingly, support from some health care professionals [29]. As concluded by Michael Saag, University of Alabama, in Birmingham, in a JAMA editorial published in December 2020 [40]:

“The clear, unambiguous, and compelling lesson from the hydroxychloroquine story for the medical community and the public is that science and politics do not mix. Science, by definition, requires diligence and an honest assessment of findings, politics not so much. The number of articles in the peer-reviewed literature over the last several months that have consistently and convincingly demonstrated the lack of efficacy of a highly hyped “cure” for COVID-19 represent the consequence of the irresponsible infusion of politics into the world of scientific evidence and discourse. For other potential therapies or interventions for COVID-19 (or any other diseases), this should not happen again”. Table 1 provides a summary of some of the key references highlighting the controversy over the use of hydroxychloroquine for the treatment of COVID-19.

## 4. Ivermectin

Ivermectin, structurally a derivative of the avermectin family of lactones, was developed in the 1970s by Merck Pharmaceuticals and used extensively from 1981 as a broad-spectrum drug to treat parasitic infections in animals. Following tests by the WHO, it was approved in 1987 for use in humans to treat river blindness (onchocerciasis) caused by a parasitic worm, *Onchocerca volvulus*. Additional uses of ivermectin followed, including treatment of parasitic infections in humans including head lice and scabies. Primarily because of its effectiveness against river blindness ivermectin was proclaimed a ‘wonder drug’ and in 2015 the Nobel Prize in Physiology or Medicine was awarded jointly to Satoshi Omura for his pioneering work in the isolation of ivermectin-related avermectins from *Streptomyces avermitilis*, and William Campbell of Merck for his work in the development and ultimate approval of ivermectin. The attractiveness of ivermectin lies in its oral effectiveness and low toxicity in humans. Its effectiveness against parasites results from its selective toxicity against glutamate-gated chloride channels in muscle and nerve [43]. Ivermectin is safe in humans at the doses used for parasitic infections (300 μg/kg) [44]; however, as with hydroxychloroquine, ivermectin is metabolized by cytochrome P450 enzymes, so there is the potential for cardiac toxicity such as *torsades de pointes* if ivermectin is used in combination with another drug that inhibits cytochrome P450 enzymes [45].

Interest in the potential for re-purposing ivermectin for COVID-19 was first raised by a report in early April 2020 by Australian scientists entitled, “The FDA-approved drug ivermectin inhibits the replication of SARS-CoV-2 in vitro” [46]. It is possible that the inclusion of “FDA-approved” in the title inadvertently heightened the expectation that the drug would prove to be effective not only in patients with COVID-19 but also for prophylactic use. The FDA and WHO both warned against the off-label use of ivermectin for COVID-19 (see [47] for example); however, their messages were essentially disregarded, and its use increased dramatically. As with hydroxychloroquine, the effectiveness of ivermectin against the SARS-CoV-2 virus was based on an in vitro cell culture protocol in which Vero/hSLAM cells were infected with the SARS-CoV-2 virus, treated with ivermectin for 24–48 h, and changes in proliferation rates determined. The data generated an IC_50_ of 2.2–2.8 nM [46], and undoubtedly this low nanomolar range made ivermectin appear to be a high-profile candidate drug for use in humans infected with SARS-CoV-2, particularly since the use of ivermectin for parasitic infections was also generally associated with low toxicity. However, a number of earlier studies had also demonstrated in vitro antiviral activity against several viruses including dengue and human immunodeficiency virus but with IC_50_ values for the importin α/β nuclear import in a low micromolar range of 18–25 µM: as concluded by the authors, the study “is not in any way proposing that ivermectin should be used at 25 µM, or anything near that to treat viral disease” [48].

A brief review of the pharmacokinetics of ivermectin also reveals that there should have been reason to suspect that even the nM concentrations reported to be effective in vitro by Caly et al. [46] would not necessarily be achieved in vivo without potential toxicity. For the treatment of onchocerciasis, ivermectin is used at a dose of ~150 µg/kg (6 or 12 mg tablets). Ivermectin has a volume of distribution (V_D_) of 3.1 to 3.5 L·kg^−1^, a half-life of ~18 h, and is strongly (~93%) bound to plasma proteins, thus indicating that the “free” form of ivermectin in the plasma will be much lower than might be assumed simply based on its V_D_ [49,50,51]. Furthermore, ivermectin has a pKa of 6.5 and therefore (unlike hydroxychloroquine) is unlikely to accumulate in lysosomes and negatively affect the acidification process for the activation of viruses. In addition, the difficulty with translating data for ivermectin from an in vitro study to therapeutic efficacy as an antiviral drug for COVID-19 was eloquently pointed out in a letter to the editor that appeared in 2021 in the *British Journal of Clinical Pharmacology* [52] showing that in a dose range from 250 to 1750 µg/kg it would not be possible for the concentration of free (unbound to plasma proteins) ivermectin in the blood to reach the level required to have an antiviral action against SARS-CoV-2. What perhaps had been overlooked in the extrapolation from the in vitro data was that is that only the free unbound ivermectin can cross cell membranes. Collectively, these pharmacokinetic data argue against the likelihood that ivermectin would prove to be an effective antiviral drug when used clinically—at least within a dose-range that would avoid significant toxicity to the patient.

**Figure 3:**
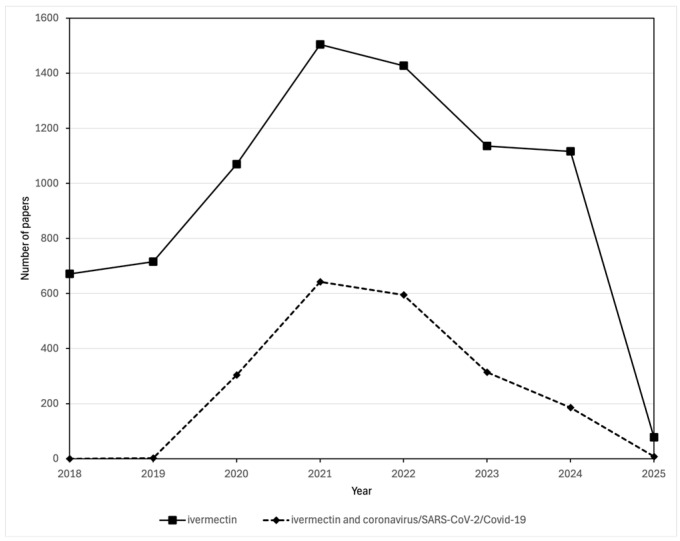
Comparison of the number of publications in the Scopus database about ivermectin and those about both ivermectin and coronavirus/SARS-CoV-2/COVID-19. Publications mentioning ivermectin were found by searching for *(TITLE-ABS-KEY (ivermectin OR ivomec OR mectizan OR stromectol)) AND (PUBYEAR > 2017)*. Publications mentioning both ivermectin and coronavirus/SARS-CoV-2/COVID-19 papers were found by searching for *(TITLE-ABS-KEY (ivermectin OR ivomec OR mectizan OR stromectol)) AND (TITLE-ABS-KEY (coronavirus OR COVID-19 OR SARS-CoV-1 OR SARS-CoV-2)) AND (PUBYEAR > 2017))*.

Despite the lack of clinical evidence that ivermectin was effective against COVID-19, considerable worldwide interest in using the drug was generated; an increase in the number of scientific publications about the drug appears to have been driven by its use in relation to the pandemic (Figure 3). And, as with hydroxychloroquine, its use became highly politicized [53]. By June 2020 as many as eight Latin American countries (Bolivia, Brazil, El Salvador, Guatemala, Honduras, Mexico, Panama and Peru) were distributing ivermectin in kits to treat COVID-19 [54,55]. In some instances, the kits were distributed not only to SARS-CoV-2 positive patients but also to households and included not just ivermectin but also hydroxychloroquine and azithromycin [55]. In July 2020, Peru distributed more than 100,000 COVID-19 kits, and in total in some countries over a million such kits were distributed, while several prime ministers and health ministers of the same eight Latin American countries also provided either direct or tacit endorsement for the use of ivermectin and hydroxychloroquine [55]. Elsewhere, prescriptions for ivermectin dramatically increased, for instance in the USA, where they rose nationally from a pre-pandemic level of 3589/week to >39,000/week in early April 2021, a 989% increase. January 2021 saw an estimated peak rate of over 140 per 100,000 people in Texas obtaining pharmacy-dispensed ivermectin [56].

Supporters for the use of ivermectin to treat COVID-19 were many, including physicians such as those belonging to the “Front-Line COVID-19 Critical Care Alliance” (FLCCC) [57]. Yet, even in 2021 a systematic review based on data from only randomized controlled studies comparing the use of ivermectin to standard care in the Cochrane Database concluded that the evidence did not support the use of ivermectin for either the prevention or treatment of COVID-19 [58]. Subsequent investigations have shown that support for the use of ivermectin was frequently based on data from uncontrolled observational studies [59,60], and potentially fraudulent studies, such as those published in predatory journals [61]: in early 2025, *Retraction Watch* reported the twelfth ivermectin-related retraction, from a then total of 467 COVID-19-related retractions [62]. Hindsight perhaps comes from a 2023 review that screened 1499 articles obtained from electronic databases and an additional two from a manual search but found only eight eligible studies (four randomized-controlled studies and four cohort studies) [63]. Based on these, the authors concluded that the low quality of the evidence precluded making a recommendation regarding the therapeutic efficacy for either the treatment or prophylaxis of COVID-19. Also of note, MedPage reported in early 2025 that the American Board of Internal Medicine has revoked the board certification of three physicians who promoted unfounded claims about COVID-19 [64]. Furthermore, the results of a randomized controlled placebo-driven study in 1432 patients with COVID-19 that was published in 2023 in JAMA showed that treatment with a maximum targeted dose of 600 µg/kg/day ivermectin had no benefit on hospital recovery time [65].

The uncontrolled, unregulated, and arguably unethical use of ivermectin effectively negated the possibility of conducting appropriately designed evidence-based clinical trials [66]. On the basis of the level of public interest in ivermectin as a cure for COVID-19, Bharti and Sismondo [12] have labelled it a “populist drug”. In contrast, the same authors called remdesivir an “establishment drug” because of the combined strong support of the pharmaceutical company, Gilead, with the USA National Institute of Allergy and Infectious Diseases (NIAID). The evidence for remdesivir is in the next section.

Unregulated use of ivermectin by the public occurred with or without prescriptions provided by healthcare professionals including veterinarians. Not surprisingly, this resulted in many instances of poisoning, for instance as detailed in September 2021 by physicians from the Oregon Health & Science University in Portland, Oregon [67]. Symptoms of poisoning included evidence of neurotoxicity, gastrointestinal, cardiovascular, and musculoskeletal problems that were more pronounced in those taking higher veterinary doses, and in several instances required care in an intensive care unit. Hoang et al. [68] noted that these symptoms were similar to those observed in previous studies of ivermectin. The provocatively titled Washington Post article “Doctors who touted ivermectin as COVID fix now pushing it for flu, RSV” [69] included a quote attributed to Dr. John P. Moore Weill Cornell Medical College in New York addressing the danger of extrapolating from in vitro studies to therapeutic efficacy in humans: “If you threw Coca-Cola into cell culture, you would see an antiviral effect. But you wouldn’t want to be squirting Coca-Cola up your nose against the flu and RSV” [69]. These reports emphasize the importance of the correct interpretation of protocol design and the evidence-based data that provided the basis for the approval of a drug (ivermectin) for a disease(s) [70].

In keeping with its “populist drug” nature [12], it is worth noting the role social media played in promoting the popularity of ivermectin. A study of over 7 million tweets on the social media platform then known as Twitter that mentioned ivermectin noted that most tweets came from the USA and Europe; when the authors focused on those originating in Nigeria and South Africa, they found that most were pro-ivermectin, and the data also showed a positive correlation between anti-ivermectin tweets and vaccination rate [71]. The role of social media in the inappropriate promotion of the repurposing of hydroxychloroquine and notably ivermectin to treat COVID-19 was highlighted by Schellack et al. [72], who noted that the majority of posts promoting their use came from unidentifiable sources. Importantly, health authorities need to take care in how they combat this kind of misinformation in the social media arena. For instance, on 1 September 2021, the American Medical Association, American Pharmacists Association, and American Society of Health-System Pharmacists issued a statement “strongly opposing the ordering, prescribing, or dispensing of ivermectin to prevent or treat COVID-19 outside of a clinical trial” [73]. The FDA in the USA subsequently used several social media platforms to issue short messages stating that ivermectin is for use in animals and not humans, e.g., “You are not a horse. You are not a cow. Seriously, y’all. Stop it” and “Hold your horses, y’all. Ivermectin may be trending, but it isn’t authorized or approved to treat COVID-19” [74]. However, in June 2022, two physicians filed a lawsuit claiming the FDA was interfering with their ability to practice medicine. Initially rejected, the suit was finally settled in 2024 with the FDA agreeing to delete and never republish the posts. The USA Court of Appeals stated that “The FDA is not a physician” and that “Even tweet-sized doses of personalized medical advice are beyond the FDA’s statutory authority” [75]. The story is not over and, as reported in the New York Times in late March 2025, ivermectin, again without credible scientific evidence, is being promoted as an anti-cancer drug [76]. In April 2025 a report on CNN noted that ivermectin was now available without prescription in Arkansas, Idaho, and Tennessee; undoubtedly this will result in more instances of inappropriate use and potential cases of toxicity [77]. Table 2 provides a summary of some of the key references highlighting the controversy over the use of ivermectin for the treatment of COVID-19.

## 5. Remdesivir

Remdesivir is a nucleotide adenosine analogue prodrug that undergoes bioactivation to the active alanine nucleoside triphosphate (GS-443902) that inhibits RNA-dependent RNA polymerase [78,79,80]. Unlike either hydroxychloroquine or ivermectin, there was prior clinical evidence to indicate that remdesivir possessed antiviral efficacy in humans in addition to extensive preclinical evidence [81]. The history of remdesivir dates to 2009 when the USA-based biotechnology company, Gilead, initiated a program to develop inhibitors of viral RNA-dependent RNA polymerase (RdRp) for the treatment of hepatitis C (HCV) and other RNA-dependent viruses. Based on promising pre-clinical studies in animals, remdesivir was used in a randomized clinical trial to treat Ebola in the Democratic Republic of the Congo during the 2014–2015 outbreak; however, based on mortality data, it was found inferior to the monoclonal antibodies (MAB114 and REGN-EB3) that were also used in the trial and the remdesivir arm was discontinued [81,82]. Studies dating back to 2014–2015 have provided in vitro (cell culture) and in vivo (mice and rhesus monkeys) support for effectiveness against some coronaviruses, namely SARS-CoV-1 and MERS-CoV, as well as respiratory syncytial virus (RSV) [81,83]. Furthermore, Brown et al. [83] reported that remdesivir inhibited coronavirus replication in human cell lines with sub-micromolar IC_50_s, providing support for potential therapeutic efficacy against SARS-CoV-1 and MERS-CoV. Similarly, in vitro studies in human lung cells and primary human airway epithelial cells infected with SARS-CoV-2 provided an IC_50_ value of 0.01 μM, with weaker anti-proliferative activity (IC_50_ = 1.65 μM) in Vero E6 cells infected with SARS-CoV-2 due to impaired bioactivation of the prodrug [84]. In vivo studies in mice and rhesus monkeys infected with MERS-CoV as well as mice and rhesus monkeys infected with SARS-CoV-2 provided additional evidence that remdesivir was an appropriate and potent antiviral candidate for trials in humans [85,86,87,88,89].

Remdesivir undergoes extensive first-pass metabolism, and its low bioavailability necessitates IV infusion with a loading dose of 200 mg/day, thereby limiting use to hospitalized patients [80]. Remdesivir has a V_D_ of ~45–75 L, with a plasma half-life of ~0.5 h for remdesivir, and ~26 h for the nucleoside triphosphate active metabolite (GS-443902). Remdesivir is also extensively bound to plasma proteins (~90%), although this is not the case for its metabolites (GS-441524 and GS-443902) [78,79,80,81,90,91]. Based on its apparent antiviral activity in cell-based studies, remdesivir has been promoted as a broad-spectrum antiviral drug with IC_50_ values in the low µM range for several infectious RNA viruses affecting humans, including filoviruses, flaviviruses, picornaviruses (including rhinoviruses), but not influenza A and B, or hepatitis B, D and E [92].

Clinical trials with remdesivir in patients with COVID-19 were initiated in early 2020 and the results were not always consistent; see Table 3 for a summary.

The double-blinded, placebo-controlled (RCT) adaptive COVID-19 treatment trial (ACTT-1) was sponsored by the National Institutes of Health (NIAID) in partnership with the USA-based biotechnology company Gilead and initiated on 21 February 2020. A total of 1062 patients with moderate to severe COVID-19 received either remdesivir or placebo plus ‘standard of care’ for 10 days. The results indicated that treatment with remdesivir reduced recovery from 15 days to 10 days [94]. An earlier but smaller RCT study in Wuhan, China (237 patients with COVID-19 in 10 hospitals: 158 received a loading dose of remdesivir of 200 mg/day then a 100 mg/day maintenance dose; 78 received a placebo and standard of care for 28 days) failed to show significant reductions in either time to recovery, mortality, or, significantly, reductions in viral load; the study was stopped prematurely due to adverse reactions [93]. The use of remdesivir is associated with liver enzyme elevations; this, plus the potential for kidney injury that is also associated with COVID-19, therefore required careful monitoring [99,100]. The SIMPLE trial initiated by Gilead, which compared two different treatment durations but did not compare to placebo, was conducted at 180 international sites and found that a shorter 5-day treatment was as effective a 10-day treatment in improving time to recovery [96,101]. Additionally, a higher percentage of subjects were discharged in the 5-day treatment arm (64.5%) versus those receiving the drug for 10 days (53.8%) [102]. Based on the data from the ACTT-1 trial as well as other studies that also concluded that remdesivir improved time to recovery, the FDA approved remdesivir for emergency use to treat COVID-19 on 1 May 2020. Full approval came on 22 October 2020 together with emergency use for pediatric patients with a body weight greater than 3.5 kg [78,79]. Similarly, many other countries followed and approved the use of remdesivir for patients hospitalized with COVID-19.

Concerns over the interpretation of the Gilead sponsored trials were detailed in *Science* in a news article from October 2020, appropriately titled “The ‘very, very bad look’ of remdesivir, the first FDA-approved COVID-19 drug” [103]. What was lacking in these trials was evidence that clinical improvement was linked to a reduction in viral load. Indeed, as already noted, the study in Wuhan, China, reported that treatment with remdesivir did not lower viral load [93]. The WHO SOLIDARITY randomized open-label trial of multiple drug treatments, conducted in 405 hospitals in 30 countries with a total of over 11,000 patients (2750 of whom received remdesivir) reported no differences in hospital mortality rates for remdesivir versus placebo plus standard of care [95]. Similarly, data from the DisCoVeRy trial conducted in Europe also showed no significant differences between the treatment groups and failed to show that remdesivir lowered viral RNA load, even after 29 days of treatment [98]. Several studies [104,105,106] detailed differences in the design and study populations of the SOLIDARITY and DisCoVeRy trials versus the ACTT-1 and SIMPLE trials that could have caused the differences in treatment results and argued that the use of remdesivir was justified. Regardless, the comparative lack of data on the effects of remdesivir treatment on viral load in the ACTT-1 and SIMPLE trials is another confounding issue that begs the question: *What was the basis for the improved ‘time to recovery*?

As the pandemic wore on, the question remained unanswered. A systematic review first published in 2022 [107] designed to determine whether remdesivir reduced length of stay, time to clinical improvement, need for oxygen support, and mortality, screened > 7000 records (after removing duplicates) with the final analysis based on 11 studies that included 4 RCTs, 5 observational studies, and 2 real world studies, the latter based on data from clinical practice. It concluded: “No significant improvement in terms of survival in patients treated with standard therapy (ST) ST + remdesivir versus ST alone (*p* = 0.24) was found”. However, the duration of oxygen supplementation was reduced for those receiving ST + remdesivir versus ST alone (*p* = 0.03). A Cochrane systematic review published in 2021 with a subsequent summary in 2023 [108,109] included 9 RCTs with a total of 11,218 subjects of whom 5982 had been randomized to receive remdesivir. It was concluded that remdesivir had no effect on all-cause mortality, or in hospital mortality in subjects with moderate to severe COVID-19. Two of the RCTs included in the Cochrane review [101,109] noted that due to ‘logistical issues’ [101] effects of remdesivir on viral loads were not reported. A small RCT of patients with moderate to severe COVID-19 comparing 5 days of remdesivir treatment (200 mg on day 1 and 100 mg for remaining 4 days) versus standard of care with 41 patients in each group also failed to show a beneficial effect of remdesivir, but effects on viral load were not reported [110].

**Figure 4:**
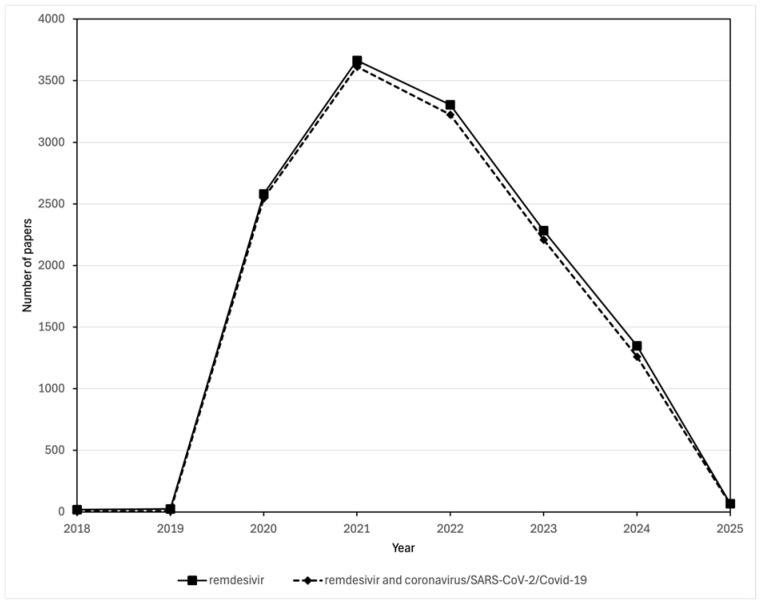
Comparison of the number of publications in the Scopus database about remdesivir and those about both remdesivir and coronavirus/SARS-CoV-2/COVID-19. Publications mentioning remdesivir were found by searching for *(TITLE-ABS-KEY (remdesivir)) AND (PUBYEAR > 2017)*). Publications mentioning both remdesivir and coronavirus/SARS-CoV-2/COVID-19 were found by searching for *((TITLE-ABS-KEY (remdesivir)) AND (TITLE-ABS-KEY (coronavirus OR COVID-19 OR SARS-CoV-1 OR SARS-CoV-2)) AND (PUBYEAR > 2017))*.

Despite, or perhaps because of, the uncertainty of its potential, the surge in studies about remdesivir that were published over the course of the pandemic almost seems to have focused almost entirely on its role in treating COVID-19. Scopus data show that the yearly number of publications discussing both remdesivir and SARS-CoV-2 throughout the period 2018–2025 were essentially the same as those discussing remdesivir alone (Figure 4). Whatever the benefits of remdesivir in treating COVID-19 might be, the political as opposed to popular nature of its support among regulatory bodies, combined with its cost of nearly $3000 for a 5-day treatment being covered for those with a USA government insurance plan, prompted Bharti and Sismondo [12] to describe it as the “establishment drug” for the treatment of COVID-19. As a result of the results of the EPIC-HR trial Paxlovid*^R^*, an orally effective combination of two anti-viral protease inhibitors, the SARS-CoV-2 inhibitor nirmatrelvir plus the HIV-protease inhibitor ritonavir, became the preferred drug treatment for outpatients with benefits shown when taken with 3 days of infection. Paxlovid also saw extensive use in hospitalized patients with COVID-19 [111,112]. Paxlovid was granted Emergency Use Authorization in the USA on 25 May 2023. The EPIC-HR (Evaluation of Protease Inhibition for COVID-19 in High-Risk Patients), was a randomized, double-blinded study of non-hospitalized adult patients with confirmed SARS-CoV-2 who were at high risk of progressing to severe COVID-19. The study compared data from 2246 adults in North and South America, Europe, Africa, and Asia with the data released by Pfizer on 14 December 2021 [113].

## 6. Conclusions

The use of what were ineffective and, in the case of hydroxychloroquine and ivermectin, potentially toxic drugs to treat millions of patients was driven by a number of factors, including the following: inappropriate extrapolation of data from in vitro studies to potential therapeutic efficacy; reliance on data from inadequately peer-reviewed publications including those published in predatory journals; information based on poorly designed clinical trials and even fraudulent data; conspiracy theories spread via social media; and overt politicization during the pandemic [12,60,114,115,116].

Collectively, as detailed in a preprint by Ricotta et al. [115], these pressures resulted in the dominance of ‘unscientific’ opinions over evidence-based medical advice from qualified healthcare experts that led to the uptake of misinformation spread by social media to a susceptible public. In some countries, but particularly well-documented for the USA [116], some government figures and news networks questioned the medical evidence that supported the seriousness of the pandemic and, indeed, argued against the healthcare policies that were initiated, including the wearing of masks, lockdowns and vaccinations. In addition, support from a number of political leaders, notably from South America and the USA, also helped fuel public demand for hydroxychloroquine and ivermectin. However, concerns were not limited to hydroxychloroquine and ivermectin; there is ongoing controversy over the effectiveness of remdesivir, the use of which was promoted with both public health and political support as the “only effective drug”, despite the negative results of the WHO-supported SOLIDARITY study—data which were released in mid-October 2020 [12,117,118]. Another negative effect of the extensive off-label use of hydroxychloroquine and ivermectin was the subsequent difficulty in conducting multi-site, international, randomized, placebo-driven, best of care clinical trials with ivermectin and hydroxychloroquine. In some countries (for example, see Requejo Domínguez et al. [55] regarding Latin American countries), inappropriate endorsement by senior government officials, including health ministers, resulted in the erosion of public trust, not only in governments, but also in science. There is an urgent need to reverse public opinion as well as government policies, enhance more transparent messaging from public health officials, and restore public confidence in the acceptance of evidence-based medicine as the source of recommendations on healthcare matters. As discussed by Hagiopol and Leru [119], the growth in size of the global scientific community has been very rapid, and although that provides benefits in terms of the rate of scientific progress, unfortunately at the same time this growth has been accompanied by increasing ethical concerns and fraud amongst scientists. They conclude: “In the post-truth era, the entire scientific community must mobilize and redouble efforts to accumulate robust evidence for scientific truth, which remains the only defendable truth” [119]. In a further development, the appearance of undeclared or inappropriate AI (ChatGPT)-generated text in the peer-reviewed literature, particularly in the fields of medicine and computer science [120], is another concern that could further erode public faith in science. Although there are benefits to the use of AI, transparency is required and appropriate fact checking essential.

Since the pandemic, work to find effective therapeutic options for SARS-CoV-2 has continued [121]. But this tale of three drugs leaves us with a clear message: we must restore public faith in science, but we also must curb the negative effects of unqualified opinions on medical and scientific issues, which are unfortunately also increasingly coming from politicians. As was the case with COVID-19, these opinions may be spread by all forms of the media, especially increasingly unregulated social media [122] and endorsement by politicians who may not possess the appropriate scientific training. Misinformation spread by multiple routes combined with a social vulnerability to unqualified opinions coming from numerous sources including the mainstream media, social-media pundits, and also politicians no doubt contributed to the widespread interest and use of risky, ineffective, and potentially dangerous treatments for COVID-19. Public announcements should not rely on personal opinions and endorsements but should instead be delegated to qualified healthcare professionals and scientists and not be subject to political interference. Even before COVID-19, misinformation on science was a serious issue as evident, for instance, with vaccine hesitancy and the link to a surge of measles cases that is also occurring again in early 2025 in Texas that have resulted in deaths and many children hospitalized [123,124]. Concerns over the re-emergence of measles, the most contagious of vaccine-preventable diseases, is not limited to the USA; the European Centre for Disease Prevention and Control reported over 35,000 cases in 2024, a 10-fold increase in the numbers for 2023, with the majority, over 30,000, in Romania [125]. Furthermore, the reductions in the funding and staffing of regulatory and research funding agencies, including the FDA and the NIH, announced in the USA in early 2025 will undoubtedly result in negative effects on efforts to prepare for the next pandemic and, indeed, hamper advances in all areas of medical care and disease prevention, as well as have a negative global impact. Collectively, these concerns were clearly expressed by the former NIH Director, Dr. Francis Collins, in an article in MedPage Today on 1 May 2025, under the heading, “*Mixing Science and Politics Leads to Trouble, Former NIH Director Says*” [126]. In the same article Dr. Collins noted the number of unnecessary deaths because of COVID-19 based on estimates by the Kaiser Family Foundation and stated “*that from June 2021 through March of 2022, 234,000 Americans died unnecessarily… That’s like three jumbo jets crashing every day for 9 months. That is the most unbelievably heartbreaking story, and that happened in the United States of America because politics and science got all tangled up*” [126].

To move forward and be better prepared not only for the next pandemic, which inevitably will come, but also to meet not just medical but other challenges such as climate changes we leave readers with the following broad recommendations:

1. Support for education programs at schools and universities that stress the importance of evidence-derived from appropriately designed and statistically evaluated research investigations that are peer-reviewed and published in reputable journals.
2. Enforce high standards and transparency for the peer-review of scientific manuscripts and, when necessary, faster retraction of published papers that for whatever reason are found to be inaccurate. In this regard *Retraction Watch* provides a very valuable service.
3. Enhance collaboration between the media, politicians and medical/scientific experts so as to ensure that public announcements are based on sound and evidence-based data and are accurately presented to the public.

## Figures and Tables

**Table 1:** Summary of selected preclinical studies and clinical trials with hydroxychloroquine for the treatment of COVID-19.

Study/Trial & Authors	Study/Trial Design	Study Details	Results	Comments & Controversies
Colson et al., 2020 [23].	Summary of published results of in vitro studies using cell lines and effectiveness of chloroquine, hydroxychloroquine (HCQ) as an anti-viral drug.	Data from 9 in vitro studies of effectiveness of chloroquine and HCQ against SARS-CoV, MERS, and other viruses.	EC50s versus SARS-CoV and MERS in the low 3–9 µM range.	Authors conclude that the results from the in vitro data suggest that HCQ as first choice to treat patients with SARS-CoV-2.
Gautret et al., 2020 [25].	Open-label non-randomized clinical trial with HCQ (200 mg tid) vs. HCQ + azithromycin (AZ) for 10 days (AZ 500 mg day1 then 250 mg for additional 4 days with ECG monitoring). Note: originally 26 patients treated with HCQ but 6 were dropped with one dying and 3 sent to ICU.	Results from 20 patients treated with HCQ or HCQ + AZ vs. 16 untreated patients from another centre, or patients refusing protocol as controls.	Effectiveness of treatment based on reduction in viral load (*‘virologically cured’*) HCQ + AZ = 100%; HCQ alone = 57.1%; control = 12.5%.	Non-randomized with very small sample size and controls not matched and concerns over the 6 dropped from study skewing the data in favour of HCQ. Accepted 1 day after submission–17 March 2020 and published on 24 March. Cited 3908 times prior to retraction.
Gautret et al., 2025 [32].	N/A	N/A (retracted paper–see [25])	N/A	Gautret et al., 2020 paper [25] -based on serious concerns over design of study and rapid review and acceptance paper was retracted on 17 December 2024.
Juurlink, 2020 [27].	Commentary on safety concerns over potential of using HCQ or chloroquine + AZ to treat COVID-19.	Literature review focusing on toxicity, drug interactions, and safety concerns with the potential for serious cardiac arrythmia due to long QT interval.	The author stresses that the Gautret et al. publication resulted in unprecedented publicity including on 21 March 2020, President Donald Trump tweeting that the drug combination as having “*… a real chance of being one of the biggest game-changers* *in the history of medicine*” [27].	Supportive evidence for the use of HCQ has been primarily derived from in vitro studies with only minimal and controversial clinical data (see [25]). Demand for HCQ to treat COVID-19 also reduces supply for patients with lupus and rheumatoid arthritis see also [40].
Lagier, et al., 2022 [24].	A retrospective analysis of 2011 COVID-19 PCR-proven infection cases at the ICH Méditerranée Infection in Marseille between March and December 2020.	Analysis of 6-week mortality of SARS-CoV-2 hospitalized patients treated with a “standard” protocol of HCQ + AX + zinc.	Treatment of COVID-19 with HCQ-AZ was associated with lower mortality, with the addition of zinc providing independent protective factor against death. No additional benefits were reported with use of corticosteroids (dexamethasone) and thus in discord with data from RECOVERY trial [41,42].	Analysis based on observational non-randomized studies with unclear criteria as to comparison with controls.
Axfors, et al., 2022 [30].	Meta analysis of mortality resulting from use of HCQ and chloroquine to treat patients with COVID-19.	Data from 14 publications/preprints with 9011 patients plus data from 1308 patients from 14 unpublished trials.	Use of HCQ was associated with increased mortality with potential for longer hospital stays associated with death (see [34]), and no benefit for chloroquine.	Highly variable dose of HCQ (400 to 1200 mg/day) and length of treatment (5 to 14 days) in the different trials.

**Table 2:** Summary of selected preclinical studies and clinical trials with ivermectin for the treatment of COVID-19.

Study/Trial & Authors	Study/Trial Design	Study Details	Results	Comments & Controversies
Caly et al., 2020 [46].	In vitro cell culture study effects of ivermectin on SARS-CoV-2.	Vero/SLAM cells infected with SARS-CoV-2 and assessment of viral RNA after 24 and 48 h with different concentrations of ivermectin.	Results show that IC_50_ for ivermectin to decrease viral RNA in low µM range (2.4 to 2.8 µM) with a ~5000-fold reduction at 48 h.	E-Pub on 3 April 2020 with provocative title, “*The FDA-approved drug ivermectin inhibits the replication of SARS-CoV-2 in vitro*”.
Peña-Silva et al., 2021 [52].	Pharmacokinetic analysis of ivermectin.	Analysis of the free (non-plasma protein bound) levels of ivermectin based on published pharmacokinetic data of use of ivermectin in humans.	Based on a range of doses of ivermectin from 250 to 1750 µg/Kg data shows that the maximum free Cmax plasma levels only marginally exceed 0.01 µM.	Estimates of free-ivermectin in plasma of humans are >100-fold lower than IC_50_ values reported by Caly et al. [46] for anti-viral effects of ivermectin in Vero/hSLAM cells infected with SARS-CoV-2. Stresses importance of understanding pharmacokinetic properties of the drug.
Popp et al., 2020 [58].	Systematic review Cochrane Data Base of randomized-control trials (RCTs) with ivermectin for treatment of COVID-19.	Data from 11 trials with 3409 patients. 8 trials were double-blinded, placebo-driven, and 3 were open label.	Based on therapeutic efficacy of ivermectin plus standard care versus standard of care plus/minus placebo alone. Analysis did not include studies on the prophylactic potential of ivermectin to reduce/prevent infection.	Authors conclude with low to high certainty that there is no evidence that ivermectin was an effective treatment for COVID-19 and nor was their evidence for a reduction in viral RNA load. None of the trials included data on the therapeutic efficacy to prevent infection, and none compared ivermectin to an effective intervention.
Kory et al., 2021 [60].	Meta analysis of RCTs studying the therapeutic efficacy of ivermectin for the prevention and treatment of COVID-19.	Summary of data included data from 18 RCTs (7 were double-blinded) and also 11 Observational Controlled Trials (OCTs).	Authors report that ivermectin use resulted in statistically significant reductions in mortality, clinical recovery, and viral clearance. In addition, data indicates that ivermectin has prophylactic efficacy to prevent infections.	Eight of the reported RCTs appeared in preprints and had not been peer-reviewed. Several were unpublished with data from www.clinicaltrials.gov (accessed on 7 March 2025).
Hu et al., 2023 [63].	Systematic review and meta -analysis of effectiveness of ivermectin in the prevention of COVID-19.	Data from 4 RCTs and 4 cohort studies were pooled. Authors assessed reliability by applying the Cochrane Risk of Bias 2.0 tool and Newcastle-Ottawa scale for RCT and cohort studies.	Analysis inferred a positive benefit of using ivermectin to protect against COVID-19, but confidence in the data was low.	The authors concluded that prophylactic ivermectin did not prevent post-exposure infection with SARS-CoV-2. For pre-exposure prevention the poor quality of the studies precluded a positive conclusion.
Naggie et al., 2023 [65].	Double-blinded RCT versus placebo with ivermectin treatment for 6 days.	1432 patients from 93 sites in USA with confirmed COVID-19 with 708 treated for 6 days with 600 µg/Kg ivermectin. Patients were followed from 16 February 2022, through 22 July 2022, and follow-up to 10 November 2022.	Six of the 21 patients were hospitalized for toxic effects from ivermectin use for prevention. Four were admitted to ICU. Symptoms included CNS (including seizures), CV and GI.	Although no deaths were reported the report illustrates the dangers of inappropriate use of an unproven drug (ivermectin).
Temple et al., 2021 [67].	Letter to editor of NEJM re. ivermectin use and toxicity reports.	Data from 21 calls in August 2021 to the Oregon Poison Control. 17 callers had obtained ivermectin from veterinary sources, and 3 received prescriptions from either physicians or veterinarians.	Six of the 21 patients were hospitalized for toxic effects from ivermectin use for prevention. Four were admitted to ICU. Symptoms included CNS (including seizures), CV and GI.	Although no deaths were reported the report illustrates the dangers of inappropriate use of an unproven drug (ivermectin).

**Table 3:** Summary of clinical trials with remdesivir for the treatment of COVID-19.

Trial & Authors	Trial Design	Number of Subjects	Results	Comments & Controversies
Wang et al., 2020 [93]. Conducted in 10 hospitals in Wuhan, Hubei, China.	Randomized, double-blinded, placebo controlled, multicentre. Loading dose of 200 mg remdesivir on day 1; 100 mg maintenance. Gilead provided remdesivir.	237: 158 to remdesivir; 79 to placebo.	Primary outcome–Time to Recovery: Use of remdesivir was not associated with significant clinical benefits. No difference in time to recovery. No difference in viral load after 28 days. Remdesivir administration was stopped prematurely due to adverse effects (GI, liver, worsening cardiopulmonary status).	Patients were permitted lopinavir-ritonavir, interferons, and corticosteroids.
Beigel et al., 2020 [94]. ACTT-1 (Adaptive COVID-19 Treatment Trial).	Randomized, double-blinded, placebo controlled, multicentre. 60 trial sites (45 in USA, remainder in UK, Greece, Germany, Korea, Mexico, Spain, Japan & Singapore). Loading dose of 200 mg remdesivir on day 1; 100 mg maintenance. Patients assessed daily from Day 1 to Day 29. Gilead provided remdesivir.	1062: 541 to remdesivir; 521 to placebo	Primary outcome: Remdesivir treatment vs. placebo reduced recovery time from 15 to 10 days. Adverse events were higher in placebo group and no deaths were attributed to treatment. Effects of remdesivir on viral load were not reported (or not studied?).	Trial protocol changed from outcome after Day 15 to outcome after Day 29. See also comments by Doggrell, 2020 [95].
Goldman et al., 2020 [96]. The SIMPLE Trial. Study supported by Gilead Sciences.	Open label and randomized to receive IV remdesivir for either 5 or 10 days. Not placebo controlled. Loading dose of 200 mg remdesivir on day 1; 100 mg maintenance.	397: 200 treated for 5 days; 197 treated for 10 days.	No significant difference in clinical outcomes were noted between 5-day versus 10-day treatment. Higher frequency of side effects (liver, kidney) in 10-day versus 5-day group, but only 44% of 10-day group completed the study due to recovery of those leaving the trial.	Interpretation limited as study was not randomized and no control. See also comments from Doggrell, 2020 [95].
Who Solidarity Trial Consortium 2022 [97]. WHO SOLIDARITY Trial.	Open-label with randomized to receive remdesivir (2750 subjects), hydroxychloroquine (954), 1411 (lopinavir without interferon), 2063 to interferon with 651 also receiving lopinavir. 4088 to placebo arm of trial.	11,330 adult subjects in 405 hospitals in 30 countries. Remdesivir treatment regimen was the same as for the ACTT-1 and Wang et al. study.	Primary outcome was in-hospital mortality with Kaplan-Meier mortality assessed at 28 days. No drug intervention resulted in a significant change in overall mortality, initiation of ventilation, or hospitalization duration.	The primary outcome for the SOLIDARITY Trial was in-hospital mortality versus recovery time for the ACTT-1 trial.
Ader et al., 2022 [98]. DisCoVeRy Trial	Open-label, Phase 3, adaptive multicentre RCT conducted at 48 sites in Europe. Patients were randomized to receive standard of care alone, or in combination with remdesivir, lopinavir-ritonavir, lopinavir-ritonavir + interferon beta-1a, or hydroxychloroquine.	Trial ran from 22 March 2020 until 21 January 2021 with 857 subjects enrolled. Remdesivir treatment regimen was the same as for the Wang et al., ACTT-1 and SOLIDARITY studies.	Primary outcome measure was clinical status at 15 as measured by WHO seven-point ordinal scale. No significant difference shown between treatment groups. The use of remdesivir was not associated with clinical improvement at day 15 or day 29, nor did remdesivir reduce mortality or lower SARS-CoV-2 RNA load.	Differences to data from ACTT-1 may relate to differences in the study population and severity of disease, with the use of corticosteroids lower in the ACTT-1 group at 23% versus 40% in DisCoVeRy.

## Data Availability

No new data were created or analyzed in this study. Data sharing is not applicable to this article.

## Abbreviations

The following abbreviations are used in this manuscript:

CDC	Centers for Disease Control and Prevention
FDA	U.S Food & Drug Administration
GISAID	Global Initiative on Sharing All Influenza Data
ICH	Institut Hospitalo-Universitaire
NIAID	U.S. National Institute of Allergy and Infectious Diseases
RSV	Respiratory syncytial virus
WHO	World Health Organization
